# Developmental Language Disorder: Early Predictors, Age for the Diagnosis, and Diagnostic Tools. A Scoping Review

**DOI:** 10.3390/brainsci11050654

**Published:** 2021-05-17

**Authors:** Alessandra Sansavini, Maria Elena Favilla, Maria Teresa Guasti, Andrea Marini, Stefania Millepiedi, Maria Valeria Di Martino, Simona Vecchi, Nadia Battajon, Laura Bertolo, Olga Capirci, Barbara Carretti, Maria Paola Colatei, Cristina Frioni, Luigi Marotta, Sara Massa, Letizia Michelazzo, Chiara Pecini, Silvia Piazzalunga, Manuela Pieretti, Pasquale Rinaldi, Renata Salvadorini, Cristiano Termine, Mariagrazia Zuccarini, Simonetta D’Amico, Anna Giulia De Cagno, Maria Chiara Levorato, Tiziana Rossetto, Maria Luisa Lorusso

**Affiliations:** 1 Department of Psychology “Renzo Canestrari”, University of Bologna, Viale Berti Pichat 5, 40127 Bologna, Italy; mariagrazia.zuccarini@unibo.it; 2 CLASTA—Communication & Language Acquisition Studies in Typical & Atypical Population, Piazza Epiro 12D, 00183 Roma, Italy; simonetta.damico@univaq.it (S.D.); chiara.levorato@unipd.it (M.C.L.); 3 Department of Education and Human Sciences, University of Modena and Reggio Emilia, Viale Allegri 9, 42121 Reggio Emilia, Italy; elena.favilla@unimore.it; 4 Department of Psychology, University Milano-Bicocca, Piazza Ateneo Nuovo 1, 20121 Milano, Italy; mariateresa.guasti@unimib.it; 5 Department of Language and Literatures, Communication, Education and Society, Università di Udine, Via Margreth, 3, 33100 Udine, Italy; andrea.marini@uniud.it; 6 Scientific Institute IRCCS “Eugenio Medea”, San Vito al Tagliamento, 33078 Pordenone, Italy; 7 Department of Mental Health, ATNO, Via Cocchi, 7/9, 56121 Pisa, Italy; stefania.millepiedi@unibo.it; 8 Health Professions Integrated Service, Azienda Ospedaliera dei Colli di Napoli, 80131 Napoli, Italy; valeria.dimartino@ospedalideicolli.it; 9 Department of Epidemiology, Lazio Regional Health Service, Via Cristoforo Colombo, 112, 00154 Rome, Italy; s.vecchi@deplazio.it; 10 Neonatal Intensive Care Unit Mother and Child Department, Ca’ Foncello Hospital, Via Sant’Ambrogio di Fiera, 37, 31100 Treviso, Italy; nadia.battajon@aulss2.veneto.it; 11 AIRIPA Associazione Italiana per la Ricerca e l’Intervento nella Psicopatologia dell’Apprendimento, Via Astichello, 10, 35135, Padova, Galleria G. Berchet, 3, 35131 Padova, Italy; laura.bertolo7@gmail.com; 12 Institute of Cognitive Sciences and Technologies, National Research Council, Via Nomentana 56, 00161 Rome, Italy; olga.capirci@istc.cnr.it (O.C.); pasquale.rinaldi@istc.cnr.it (P.R.); 13 Department of General Psychology, University of Padova, Via Venezia 8, 35131 Padova, Italy; barbara.carretti@unipd.it; 14 Department of Clinical Medicine, Public Health, Life and Environmental Sciences, University of L’Aquila, Palazzo Camponeschi, Piazza Santa Margherita 2, 67100 L’Aquila, Italy; mariapaola.colatei@univaq.it; 15 Studio di Psicoterapia e Riabilitazione dell’età evolutiva, Via Annone 1, 00199 Roma, Italy; cristina.frioni@gmail.com; 16 Department of Intensive and Robotic Neurorehabilitation, IRCCS Bambino Gesù Children’s Hospital, Lungomare Marconi 36, 00058 S. Marinella, Roma, Italy; luigi.marotta@opbg.net; 17 Azienda Usl Toscana Centro, Piazza Santa Maria Nuova 1, 50121 Firenze, Italy; sara.massa@uslcentro.toscana.it; 18 Federazione Logopedisti Italiani, Via Daniello Bartoli, 00152 Roma, Italy; letizia.michelazzo@gmail.com (L.M.); manupieretti@gmail.com (M.P.); annagiulia.decagno@gmail.com (A.G.D.C.); tizzi.ross@gmail.com (T.R.); 19 Department of Education, Languages, Intercultures, Literatures and Psychology, University of Florence, Complesso di San Salvi, Padiglione 26, Via di San Salvi 12, 50135 Firenze, Italy; chiara.pecini@unifi.it; 20 Department of Biomedical and Clinical Sciences “L. Sacco”, Università degli Studi di Milano, Via G.B.Grassi, 74, 20157 Milano, Italy; silvia.piazzalunga@unimi.it; 21 UO Neurology and Neurorehabilitation, IRCCS Stella Maris Foundation, Viale del Tirreno 331, 56128 Calambrone, Italy; renata.salvadorini@fsm.unipi.it; 22 Department of Medicine and Surgery, University of Insubria, Via Ravasi 2, 21100 Varese, Italy; cristiano.termine@uninsubria.it; 23 Biotechnological and Applied Clinical Sciences, University of L’Aquila, P.le S. Tommasi, 1, 67100 Coppito, Italy; 24 Department of Developmental Psychology and Socialization, University of Padova, Via Venezia 8, 35131 Padova, Italy; 25 Department of Child Psychopathology, Scientific Institute IRCCS E. Medea, Via Don Luigi Monza 20, 23842 Bosisio Parini, Italy; marialuisa.lorusso@lanostrafamiglia.it

**Keywords:** developmental language disorder, late talkers, language delay, early predictors, screening tools, diagnostic tools, age of assessment, evidence-based medicine

## Abstract

Background. Developmental Language Disorder (DLD) is frequent in childhood and may have long-term sequelae. By employing an evidence-based approach, this scoping review aims at identifying (a) early predictors of DLD; (b) the optimal age range for the use of screening and diagnostic tools; (c) effective diagnostic tools in preschool children. Methods. We considered systematic reviews, meta-analyses, and primary observational studies with control groups on predictive, sensitivity and specificity values of screening and diagnostic tools and psycholinguistic measures for the assessment of DLD in preschool children. We identified 37 studies, consisting of 10 systematic reviews and 27 primary studies. Results. Delay in gesture production, receptive and/or expressive vocabulary, syntactic comprehension, or word combination up to 30 months emerged as early predictors of DLD, a family history of DLD appeared to be a major risk factor, and low socioeconomic status and environmental input were reported as risk factors with lower predictive power. Optimal time for screening is suggested between age 2 and 3, for diagnosis around age 4. Because of the high variability of sensitivity and specificity values, joint use of standardized and psycholinguistic measures is suggested to increase diagnostic accuracy. Conclusions. Monitoring risk situations and employing caregivers’ reports, clinical assessment and multiple linguistic measures are fundamental for an early identification of DLD and timely interventions.

## 1. Introduction

Language development is a complex process resulting from the interplay between biological, cognitive and environmental factors (e.g., [1,2,3]). Typically, by the age of 10–12 months, children tune in on the phonemes of their mother language and can implicitly discriminate them (for a review, see [1]). By the same age, they begin to understand and utter their first words and produce deictic and representational gestures [4,5]. Early word comprehension and gesture production are tightly associated [4,6] and predictive of expressive vocabulary at 24 months [7]. At around 18 months, children reach a lexical repertoire of approximately 50 words and produce frequent gesture–word combinations; at between 20 and 24 months, they further increase their expressive vocabulary and begin to produce two-word utterances [5,8,9]. By the age of three, they have developed a relatively rich mental lexicon and their utterances, grammatically more accurate and complex, can also be understood by people outside the familial environment (for a review, see [10]).

Nonetheless, 11–18% of children aged between 18 and 36 months may show a significantly slowed lexical development in the absence of brain lesions, intellectual disability, or hearing impairments. These children are usually labelled late talkers [11,12,13] and may present with reduced expressive and/or receptive language [14,15,16]. The large majority of late talkers show a significant lexical improvement after the age of three that allows them to perform within normal limits on linguistic tasks, even if some difficulties may persist in their daily communicative interactions [17,18,19,20].

### 1.1. Developmental Language Disorder

Some late talkers, however, will not catch up with their peers and, after the age of three, will likely receive a diagnosis of Language Disorder [21], more recently labelled Developmental Language Disorder (DLD, [22]). This diagnostic label has been introduced by the CATALIZE Consensus in order to refer to children who have a language disorder that emerges during development, not being acquired or associated with known biomedical conditions (e.g., brain injury, neurodegenerative diseases, cerebral palsy, or other difficulties related to genetic or neurological causes [22]). The CATALIZE Consensus also agreed that a diagnosis of DLD is not precluded by the presence of neurobiological or environmental risk factors or the co-occurrence with other neurodevelopmental disorders and does not require a mismatch between verbal and nonverbal ability [22]. Crucially, it is now widely accepted that these linguistic difficulties may co-occur with a variety of other cognitive weaknesses, such as in procedural memory [23], motor control [24], phonological working memory [25], and executive functions [26,27], with growing evidence about the impact of such difficulties on linguistic performance (e.g., [28]). Although terms such as ‘Primary Language Impairment’ and ‘Primary Language Disorder’ have also been used in the literature to account for the aspecificity of this language disorder and its unknown origin [29,30,31,32,33] (for further details, see the Methods section), we will use the term DLD, according to the more recent international consensus [22], regardless of how this disorder was labelled in the papers reported in this review.

DLD is a highly heterogeneous condition [22]. It can affect language production and/or comprehension with various degrees of severity in different aspects of language processing (e.g., lexical, morphosyntactic, pragmatic [21,22,34,35]; for a review, see [36]). Furthermore, it is among the most frequent developmental disorders [37] with neuropsychological sequelae in about 40–50% of cases [17]. Such sequelae may become particularly evident during the transition from oral to written language [38,39]. Affected children may show long-lasting learning problems [40,41], estimated five times higher in children with DLD than in children with typical development [42,43]. Furthermore, they may show behavioral, psychiatric, emotional, and social adaptation difficulties [44,45,46,47] that might eventually affect their working and relational skills as adults [48,49].

These considerations support the importance of an early identification of children with DLD because prompt inclusion into a rehabilitation program might help them improve their language skills by the age of 5 and reduce the risk of subsequent sequelae [50]. To achieve this goal, it is imperative to identify early predictors before the time of the diagnosis as well as potential risk factors that may lead to DLD.

### 1.2. Risk Factors and Early Predictors of Poor Language Prognosis

Growing evidence suggests that potential risk factors include a family history of speech and language impairments, a low level of parental education and/or socioeconomic status (SES), male gender, and pre- or peri-natal factors such as being born preterm or with low birth weight [19,22,51]. However, the role potentially played by such factors in screening is still unclear since many investigations considered heterogeneous populations with different types of delay or disorder. Besides risk factors, several studies have focused on identifying the early predictors of DLD, such as a limited expressive vocabulary, absence of word combinations, poor comprehension, and absence of gestures between the second and third year of life. Nonetheless, a consensus on the predictive power of these early indicators is still missing [19,20,22].

### 1.3. Effective Tests and Optimal Time to Avoid Diagnostic Bias

Another highly relevant issue for clinical purposes concerns the need to identify effective tests for language assessment in children with language impairments. The effectiveness of diagnostic tools is usually measured in terms of: (a) validity, i.e., whether a tool measures what it claims to measure; (b) accuracy, identified by productivity measures such as sensitivity (proportion of clinical cases correctly classified by the test), specificity (proportion of normal cases correctly classified by the test), Likelihood Ratio (LR = sensitivity/1-specificity), and Positive Predictive Value (PPV, proportion of screen positives that are true cases = number of true positives/number of true positives + number of false positives); and (c) reliability, i.e., the degree of stability of measurement when repeated under different conditions or by different observers. Effective tests might allow clinicians to minimize potential diagnostic biases to avoid issues of overdiagnosis (i.e., when a child, who does not have a linguistic impairment, is mistakenly identified as a child with DLD) or underdiagnosis (i.e., when a child, who does have a linguistic impairment, is mistakenly identified as a child with typical language development). In addition, for an accurate diagnosis, it is advisable to also include tasks assessing spontaneous speech with multilevel procedures of analysis that have proved highly sensitive to linguistic difficulties (e.g., [52]).

The possibility of diagnostic bias is further enhanced by another ambiguity in the available literature that concerns different cut-offs used for the diagnosis: 1 standard deviation (SD), 1.25 SDs, 1.5 SDs, or 2 SDs below the expected mean [53,54,55,56,57,58].

In addition, an optimal time for screening and diagnosis needs to be identified in order to minimize diagnostic biases.

### 1.4. Aims of the Review

The available literature clearly shows some of the most critical challenges that might undermine an accurate diagnosis of DLD in preschoolers. Coherently with these premises, we performed a scoping review to provide answers to these challenging issues concerning (a) early predictors, (b) optimal time for screening and diagnosis, and (c) effectiveness of diagnostic tools for the identification of DLD in preschool children.

## 2. Methods

We performed a systematic search of studies as part of a Consensus Conference about diagnosis and treatment of children with language disorders, held in Italy in November 2018 and published in November 2019 [32]; we further updated the search until December 2020. This Consensus Conference agreed to use the diagnostic label *Disturbo Primario del Linguaggio* (i.e., Primary Language Disorder) for the Italian context [32,33]. For the Italian clinical system, this label refers to a developmental language disorder that is not acquired or associated with a known biomedical cause. Similar to DLD [22], its diagnosis is not precluded by the presence of neurobiological or environmental risk factors or co-occurrence with other neurodevelopmental disorders and does not require a mismatch between verbal and nonverbal ability. Therefore, the notion of Primary Language Disorder, as used in this Consensus Conference [32,33], corresponds to that of the term DLD, recently adopted in the literature [22], already illustrated in the Introduction and used throughout the present review. This Consensus Conference followed the steps detailed in the Methodological Manual of the Italian Superior Institute of Health [59].

This scoping review employed the following four-stage model: (i) identifying the research question; (ii) identifying relevant studies; (iii) study selection; (iv) and (v) summarizing and reporting the results.

### 2.1. Identifying the Research Question

The experts involved in the Consensus conference focused on three clinical questions:

Q1. Are there early predictors for the identification of DLD?
Q2. What age range is most appropriate for use of screening and diagnostic tools for DLD?
Q3. What tools are effective (in terms of validity, accuracy, and reliability) for the formulation of a diagnosis of DLD in preschool children?

### 2.2. Source of Data

A broad systematic search of the relevant literature published until December 2020 was conducted by examining the following databases: PubMed, Embase, Web of Science, The Cochrane Central Register of Controlled Trials (CENTRAL; 2017 Issue 2), SpeechBITE (speechbite.com), PsycINFO (via Ovid). For each database, a search strategy was developed considering Medical Subject Headings (MESH) and free terms (see Appendix A, Table A1). Finally, experts and practitioners in the field, participants in the scientific–technical committee, and working groups of the Consensus Conference indicated further potentially relevant studies. Two independent reviewers screened titles and abstracts to exclude irrelevant studies. Potentially eligible studies were retrieved in full text and screened for eligibility. Disagreements were resolved through discussion.

### 2.3. Selection Criteria and Data Extraction

This revision included systematic reviews, health technology assessment reports, meta-analyses, and primary observational studies with control groups. Included studies evaluated the relevant early factors for predicting language difficulties or reliability and diagnostic accuracy of tools for the identification of children with DLD. Studies assessing the accuracy of screening tools for the early identification of language delay and late talkers were also included. Outcomes of interest were language outcomes, predictive values, sensitivity and specificity values of diagnostic tests, and psycholinguistic measures. The included studies investigated such issues on preschoolers. When the included studies involved both preschool and school age children, only results concerning preschool-age children were reviewed.

Studies on children with cognitive delay, deafness, autism spectrum disorders (ASD), genetic syndromes (e.g., Down syndrome, Klinefelter syndrome), neurological deficits, pervasive developmental disorders, traumatic brain injuries, primary disorders (sensory, neurological, psychiatric), dysphonia, dysarthria, dysrhythmias, stuttering, specific speech articulation disorder or dyslexia were excluded. Studies that included bilingual children were also excluded because the issue of bilingualism would have introduced too many additional variables to be accounted for such as age of acquisition, levels of proficiency, number of acquired languages, type of bilingualism [60,61] as well as issues related to language assessment [62] and the potential role of bilingualism as a risk or protective factor [63] that would deserve a dedicated review.

For each included study, three independent reviewers extracted information on the study design, population characteristics, type of test, type of comparison group, language domain assessed, and tool, setting and figures involved. For predictive studies, biological and environmental factors, variables from toddlerhood assessments, and background information assessed for predicting the risk of developing DLD were extracted. For screening and diagnostic studies, sensitivity, specificity, and positive and negative predictive values of the tool were extracted. An assessment of the quality of the studies was not performed.

### 2.4. Data synthesis

The characteristics of the included studies were reported in Table A2 and Table A3 (see Appendix B) and results were summarized narratively.

## 3. Results

### 3.1. The Selected Literature

**Figure 1:**
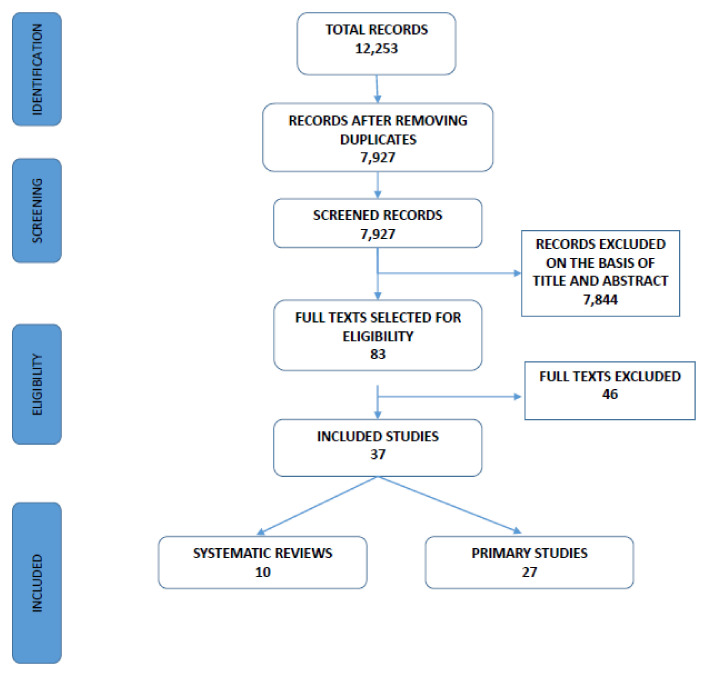
Flow diagram of the included studies.

Figure 1 shows the process of selection of the studies. Through bibliographic searches, 12,253 studies were identified. Of these, 7927 were selected after removing duplicates. Two independent reviewers judged the eligibility of the included studies based on the title and abstract. After this screening, 7844 studies were excluded. Consequently, 83 studies were retrieved in full text for a more detailed evaluation. Of these, 37 papers (10 systematic reviews and 27 primary studies) on early predictors of DLD and tools for diagnosis were included. Forty-six studies were excluded.

Results are presented according to the study design and main issues (Q1: early predictors; Q2: optimal time for screening and diagnosis; Q3: effective tools for diagnosis).

#### 3.1.1. Systematic Reviews

Table A2 in Appendix B reports on the characteristics of the 10 systematic reviews. Among the selected reviews, Law et al. [64] and Law et al. [65] considered the same studies, even if with different objectives. Moreover, the article by Wallace et al. [66] was an update of a previous study by Nelson et al. [67]. The number of included studies in these reviews ranged from 1 [68] to 45 [65]. Most of these reviews included prospective cohort studies, while some were based on cross-sectional studies. Five studies were case–control studies, and one was a randomized clinical trial (RCT).

Almost all of the systematic reviews focused on preschool age children. As stated earlier, if a review involved both preschool and school age children, only results concerning preschool age were reviewed in this study.

The languages more often considered are English and German. Other languages included are Spanish, Italian, Swedish, French, Finnish, Dutch, Slovenian, Hebrew, Farsi, Cantonese, Mandarin, Portuguese, and Norwegian. In several studies, the language of the examined sample was not specified, and in some cases, it was deduced from the description within the articles.

Six reviews [64,65,66,67,68,69] focused on issues related to the early identification of language difficulties: the diagnostic accuracy of screening tests [64,65,66,67,68,69]; the factors determining the quality of a language screening (e.g., age) [66,67]; selection of populations with major risk factors [66,67]; etiology of DLD [66]; the potential adverse consequences of screening [66,67]; the contribution of parent-rated screenings [69]; the contribution of screeners rated by pediatricians or other trained examiners [66,68].

Two reviews address the psychometric qualities of diagnostic tools. The review by Denman et al. [70] assessed the psychometric quality of 12 diagnostic spoken language tests for monolingual English-speaking children; Maleki Shahmahmood et al. [71] reviewed the studies published from 2000 to 2015 with the objective of determining the sensitivity and specificity of language tests or measures in identifying preschool children with DLD and distinguishing them from Typically Developing (TD) children.

Finally, two reviews addressed early predictors and risk factors: Fisher [72] explored the factors predicting preschool-age expressive language outcomes among late talkers. Bettio et al. [73] examined the literature in order to identify risk factors associated with delays in the development of children’s oral language, as well as protective factors that could moderate the effects of risk factors.

#### 3.1.2. Primary Studies

Table A3 in Appendix B reports on the characteristics of the 27 selected primary studies. Almost all of them were observational, with either a longitudinal or a cross-sectional design, and focused on preschoolers. If they involved both preschool and school age children, only results concerning preschool age were reviewed. Data collection was usually performed in either a clinical or research context. The studies concerned monolingual children, speaking mainly English and German, but also Afrikaans, Arabic, Catalan, Finnish, Hebrew, Korean, Icelandic, Italian, isiXhosa, Lithuanian, Luxembourgish, Norwegian, Polish, Portuguese, Serbian, Slovak, Spanish, Swedish, and Turkish. One study provided a cross-linguistic comparison among monolingual children of several languages for different versions of a test assessing lexical production and comprehension [74].

As for the identification of early predictors (Q1), some studies examined the role of biological and environmental risk factors for DLD [75,76,77,78,79] and many provided potentially useful data about early gestural, communicative, lexical, and grammatical predictors [75,76,79,80,81,82,83,84,85,86,87,88,89].

Even if none of the selected studies directly focused on the problem of the most appropriate age range for the use of screening and diagnostic tools (Q2), almost all of them provided indirect indications to answer Q2 [74,75,76,77,78,79,80,81,82,83,84,85,86,87,88,89,90,91,92,93,94,95,96,97,98,99,100].

As for the problem of validity, accuracy, and reliability of diagnostic tools for DLD (Q3), four studies examined the concurrent validity of psychometric characteristics of diagnostic tools, i.e., the Preschool Language Scale—PLS-5 [97], the Fluharty-2 test [98], the Tamiz de Problemas de Lenguaje—TPL [99] or screening tools for DLD, i.e., the Screening for Identification of Oral Language Difficulties by Preschool Teachers (SIOLD) [100].

The following sections will be devoted to the analysis of the selected literature with reference to each of the three questions addressed in the present review.

### 3.2. Q1: Are There Early Predictors for the Identification of DLD?

#### 3.2.1. Results from the Systematic Reviews

The results from the systematic reviews will be presented in two different sections, the former focusing on risk factors and early predictors (even if the information on the latter is rather limited and not always consistent across the different reviews), and the latter presenting the effectiveness of screening procedures.

##### Risk Factors and Early Predictors

With regard to the risk factors for DLD, the studies mainly reported biological factors, such as family history for DLD and male gender. In Bettio et al.’s review [73], a family history of language delay and writing and reading difficulties turned out to be reliable predictors of persistent language delay between three and five years (the authors used the term “language delay” to refer to both a pre-diagnosis late talker status and a persistent language delay that might be—albeit not necessarily—equivalent to DLD). By contrast, gender may be a predictive factor of language development in children under the age of three. After this age, gender difference might become less evident. Other factors, related to pre- and peri-natal conditions (e.g., low birth weight, prematurity, birth order, etc.) and to socioeconomic conditions (i.e., low parental educational level and/or socioeconomic status, family size, having very young or very old parents, belonging to an ethnic minority), showed associations (with expressive more than receptive measures of language in the case of birth weight [73]) that, however, do not always reach significance [66,67,73]. One of the studies reviewed by Bettio and colleagues [73] reported a persistent gap in vocabulary equivalent to eight months of vocabulary growth between children with and without socioeconomic disadvantages. Notably, two of the studies included in their review [73] support the view that environmental factors exert a higher influence on language development compared to biological/genetic ones. For instance, the effects of biological risk (low birth weight) on language development appeared to be contingent on environmental factors such as parental responsiveness. Belonging to a family with four or more children, by contrast, seemed to be a predictive factor (possibly linked to divided attention by parents) only up to the age of four. Other dynamic factors reported in Bettio et al.’s review [73] point to the importance of mother–child interaction at both a qualitative (in turn, influenced by the mother’s mental health status) and quantitative level (number of communicative interactions, time devoted to reading to the child, etc.). However, the review also suggested that the predictive value of such factors is low and that measurement issues (i.e., vocabulary measures through parental reports represented by the MacArthur–Bates Communicative Development Inventory (MB-CDI) for two of the included studies, by the Ages and Stages Questionnaires (ASQ) for two other studies and by non-standardized, ad hoc questions to the parents for an additional study) as well as overlap with other environmental factors (e.g., different language spoken at home and at kindergarten) may be confounding factors.

Relevant information on early predictors can be found in the review of Bettio and colleagues [73], showing that low receptive language at one and half years of age (measured by standardized tests or by non-standardized, ad hoc questions to the parents), along with (unspecified) nonverbal cognitive measures at three years of age, predict persistent language delays between three and five years, up to eight years.

Fisher’s review [72] investigated specifically the predictive power of expressive vocabulary, receptive language, mean length of utterance (MLU), socioeconomic status (SES), gender, family history, with respect to later expressive language outcomes in late talkers. Results of meta-analysis showed a medium correlation between expressive-vocabulary size and expressive language outcome (r = 0.249, *p* < 0.01) and between receptive language and outcome (r = 0.340, *p* < 0.01). A small significant effect was observed for socioeconomic status (r = 0.111, *p* < 0.01). MLU and male gender showed no significant effects; family history also lacked predictive power, possibly due to the heterogeneity of the type of family history assessed in the various studies.

##### Effectiveness of Screening Procedures

Regarding the effectiveness of large-scale screening procedures, no clear indications emerged in a study focusing on German-speaking children [68]. Nonetheless, the results showed some correlations with the subsequent stages of language development and no particular drawbacks were described.

Law et al. [64] assessed the quality of screenings based on productivity measures, namely on sensitivity, specificity, Likelihood Ratio, and Positive Predictive Value. They noted that, usually, the higher the LR, the better the screening; nonetheless, with very frequently occurring disorders such as DLD, they argued that, even if high values of both sensitivity and specificity are desirable, it is better to have high specificity (accurate identification of children without speech or language delays, i.e., few false positives) than high sensitivity (accurate identification of children with speech or language delays, i.e., few false negatives). Indeed, increasing specificity (more than increasing sensitivity) would maximize the number of true cases identified through the screening process, i.e., the LR parameter, and thus the cost-effectiveness of the procedure. Among the screening tools with high productivity measures that can be used before age 4, Law et al. [64] included the Clinical Linguistic and Auditory Milestone Scale (CLAMS; [101]), the Early Language Milestone Scale ([ELM; [102,103]), the Hackney Early Language Screening Test [104,105], the Language Development Survey (LDS; [106,107,108,109]), the Levett–Muir Screening Test [110], the Rigby Speech Screening Test [111], and the Screening Kit of Language Development ([SKOLD; [112]).

The study by Wallace et al. [66] confirmed the validity of some screening tools, such as, for parents, the MacArthur–Bates Communicative Development Inventory (MB-CDI; [113]), and the Infant Toddler Checklist of the Communication and Symbolic Behavior Scales ([CSBS ITC; [114]), and, for professionals, the Screening Kit of Language Development ([SKOLD, [112]) questionnaires. The fact that the review did not reveal any large differences between tools for parents and for professionals suggests that the use of tools filled out by parents (e.g., MB-CDI, CSBS ITC, and LDS) can be useful for screening without overloading health services. Indeed, according to the review by Sim et al. [69], universal screening tools for language and behavior concerns in preschool-aged children used in a community setting can demonstrate excellent predictive validity, particularly when based on parent-reported assessment. The screening tool with the best predictive value with regard to language development was the Language Development Survey (LDS [106,107,108,109]) administered at age 2 years (sensitivity 67%, specificity 94%, Negative Predictive Value NPV 88% and PPV 80%). The LDS was followed by the MB-CDI, in its toddler version forms ELFRA-2 [115] and SETK-2 [116] (sensitivity 61%, specificity 94%, NPV 95%, PPV 56%), although another study found lower figures (sensitivity 50%, specificity 67%, NPV 60.5% and PPV 58%) with the British short form (MB-CDI-UKSF) [117]. Mixed results were found also for the Strengths and Difficulties Questionnaire (SDQ) [118], the Denver Developmental Screening Test (DDST) [119,120], and the General Language Screen (GLS) [121] for which both sensitivity values (31%, 44%, and 67.4%, respectively) and PPV values (31%, 41%, and 31.5%, respectively) were particularly unsatisfactory—although they could improve when combining language and behavioral measures (e.g., SDQ and Sure Start Language Measure—SSLM, at 30 months) [122]. In general, parent report screening tools for language achieved higher sensitivity, specificity and negative predictive value than direct child assessment.

Additionally, the review by Maleki Shahmahmood et al. [71] suggests that the combination of several measures provides better results. The specific combined measures vary from study to study and from language to language, ranging from the combination of different grammatical markers or of grammatical markers and standardized tests for English-speaking and Italian-speaking children, to the combination of experimental measures and spontaneous production measures, such as mean length of utterance—MLU—in Spanish-speaking and French-speaking children. Furthermore, this study showed that 3-year-old at-risk children produce fewer communicative gestures.

Summarizing the data on the psychometric properties of the screening instruments, not all the tests have shown adequate levels of sensitivity and specificity. Usually, sensitivity is lower than specificity (even if this can vary depending on the test): early identification of children without speech or language delays is easier than the identification of children with delays. Furthermore, sensitivity tends to improve with age, whereas specificity remains high. Screenings based on speech show higher Likelihood Ratio values at lower ages, whereas screenings based on language only do at older ages [65].

In conclusion, there does not seem to be sufficient evidence for the introduction of universal screenings. Recommendations for alternative approaches to early identification of speech delays include clinical examination, confirmatory screening (in two phases: the first one based on parents’ reports, the second one on clinical assessment), primary prevention with the parents, and monitoring of risk situations [65].

#### 3.2.2. Results from the Primary Studies

Some of the primary studies examined the role of biological and environmental risk factors for DLD. The longitudinal study by Hsu and Iyer [75] showed a prevalence of DLD in males, children belonging to ethnic minorities, and born to mothers with low levels of formal education and with poor linguistic skills. Marini et al. 2017 [76] found that a family history of DLD is 2.5 times more common in late talkers than in typically developing (TD) children and that late talkers have significantly lower mean scores of home literacy environment than children with TD. Two other primary studies examined the role of biological and environmental risk factors in late talkers. As for speech production, Suttora et al. [77] identified a family history of DLD and/or learning disorders as the only significant biological risk factor. Moreover, greater lexical diversity, rate and the grammatical complexity of the parental linguistic input during parent–child book sharing were the only significant environmental risk factors for 30-month-old late talkers. Child cognitive score was positively associated with child speech production. In a longitudinal investigation focusing on late talkers, Conway et al. [78] highlighted that the participants’ language scores at 24, 36 and 48 months were positively associated with good mother–child interaction qualities (in terms of fluency and connectedness). At the same time, they were negatively associated with maternal directives in mother–child play interactions at 24 months. The latter association attenuated after adjusting for co-occurring maternal responsive expansions and was strongest for children exposed to lower quality interactions. The studies by Suttora et al. [77] and Conway et al. [78] showed the fundamental role of mother–child interaction and maternal communicative–linguistic input quality for the linguistic development of late talkers. Taken together, the findings from the above-described primary studies highlight the interaction of biological and environmental (i.e., parental and home) risk factors impacting on language trajectories of children with TD and late talkers.

Several primary studies provide potentially useful data regarding early gestural, communicative, lexical, and grammatical predictors.

As for early gesture production, Lüke et al. 2017 [79] suggested that pointing at 12 months with one finger and not with the whole hand may predict a good development of receptive and expressive language at 2 years, while its absence can be considered as a predictor of primary language delay in children. In addition, Lüke et al. 2020 [80] followed children with typical development (TD) and children with a language delay (LD) or DLD from one to six years of age with a total of 14 observations. They showed that pointing with the extended index finger at 1;0 year is predictive of future language skills up to 5;0 and 6;0 years. This predictive effect is mediated by language skills at 3;0 years and by iconic gesture comprehension at 3;0 years for grammar skills at 5;0 and 6;0. Their findings support the view of an integrated speech–gesture communication system and the importance of an early assessment of gesture production and comprehension to detect children with LD. Further evidence on the early predictive value of the pointing gesture comes from the longitudinal study by Sansavini et al. [81] that followed, at 18, 24, 30, and 36 months, children with TD and children belonging to two groups at risk for LD because of being born extremely preterm, with no neurological damage or intellectual disability, or being a sibling with no ASD of a child with ASD. Their findings showed that, in both at-risk groups, only some children exhibited LD. Interestingly, gesture production at 18 months, coded during mother–infant play interaction and, particularly, pointing gesture, was significantly lower in children later detected with LD than in children with TD, demonstrating that a low rate of pointing gesture at 18 months may be a reliable and common predictor of LD across different populations of infants with enhanced LD risk.

As for communication skills, Morgan et al. [82] showed that measures of social communication, collected with the Communication and Symbolic Behavior Scales Caregiver Questionnaire (CSBS CQ, a parent report) and Behavior Sample (CSBS BS, a clinician-administered tool) [123] between 18 and 21 months, may help predict language outcomes at 2 and 3 years as measured with the Mullen Scales of Early Learning (MSEL) [124]. Therefore, the CSBS adds significantly to the information obtained by parent-reported expressive vocabulary production, measured with the Language Development Survey (LDS) [106] at 24 months. The LDS and the speech composite scores derived by the CSBS CQ and BS were found to be significantly associated with language delay at 2 years of age, whereas the symbolic and social composites of the CSBS CQ and BS were related to language delay at age 3. These findings suggest that the early identification of children with persistent language delay should not be limited to the assessment of lexical production but should embed an analysis of the child’s communicative behavior and his/her comprehension skills with a joint use of parent reports and clinician-administered tools. As effect sizes were small overall, the authors argued that other variables, such as a family history of DLD, should also be considered for a stronger prediction of persisting language delay. Vehkavuori and Stolt [83] analyzed the specificity and sensitivity of two screening methods (i.e., the Finnish versions of the MacArthur Communicative Development Inventories Short-Form (FinCDI-SF) and the Communication and Symbolic Behavior Scales, Developmental Profile, Infant-Toddler Checklist (FinCSBS)) [125] compared to the performance on the Reynell Developmental Language Scales III—Finnish version [126] at 24 months. The two screening tools were shown to have high specificity but only moderate sensitivity. The use of word combinations and parental concern may provide further relevant information in identifying children with weak language skills. Nonetheless, they were not sufficiently accurate if not associated with an assessment of the child’s receptive and expressive skills. In line with the findings by Morgan et al. [82], this study further highlights the need for the assessment of both receptive and expressive communicative–linguistic skills in language screening at 2 years of age. The study by Kim et al. [84], on a population of Korean children with a mean age of 29.7 months, highlighted the validity of the Korean integral version of the Ages and Stages Questionnaire (K-ASQ Questionnaire; [127]) as a screening tool for mixed developmental disorders and—to a lesser degree—for isolated DLD. The K-ASQ test appears to be a good screening tool but, since the communication domain is composed of six different items exploring the children’s receptive and expressive linguistic skills, it is not possible to discriminate to which item the sensitivity of the scale is related, thus failing to provide crucial information for the question under consideration.

As for expressive vocabulary, in Hsu and Iyer [75], expressive (but not receptive) vocabulary at 15 months (assessed with the MB-CDI) contributed to DLD risk at 3 and at 4;6 years, also mediating the effects of gesture production at 15 months (again assessed with the MB-CDI). Hadley et al. [85] showed that the number of words produced by children at 24 months predicts syntactic and morphosyntactic development at 30 months. Indeed, the authors reported correlations between the MB-CDI and words in spontaneous speech (total number of different words, names, verbs) at 24 months. Assuming the 10th percentile as a threshold for at-risk status, a spoken vocabulary with fewer than 2 verbs at 24 months, 10 verbs at 27 months, and 46 verbs at 30 months can be considered as predictors. It is usually believed that using more verbs implies mastering more grammatical structures; nonetheless, from the results reported by parents in the MB-CDI, the authors found that verb production alone did not predict syntactic complexity at 30 months. Indeed, verbs and names seemed to be equally valid predictors. In Bello et al. [86], vocabulary measures, assessed with the Italian version of the MB-CDI [128] at 29 months, predicted lexical development at 34 months. In addition, a greater number of late talkers at 29 months, relative to Italian normative data, had weaknesses in gesture production, decontextualized comprehension, and verbal imitation, and did not show the ability to perform symbolic play according to parental reports. This provided further evidence that not only expressive vocabulary, but also gesture production and symbolic skills should be assessed in late talkers at this age. Kademann et al. [87] confirmed that an initial delay in expressive vocabulary (a vocabulary with fewer than 50 words at 2 years) may predict potential linguistic difficulties at 4;6 years. However, the study by Kademann et al. [87] has some limitations because the sample, as stated by the authors, is not representative of the population for socioeconomic status and educational level (higher than the average, but lower than in other longitudinal studies); furthermore, some of the children underwent speech therapy from 3 to 4 years. Marini et al. [76] assessed 293 children longitudinally at approximately 32 (t1) and 41 (t2) months. The Italian adaptation of the Language Development Survey [129] proved to be sensitive in identifying late talkers at t1. At t2, 33 children, identified as late talkers at t1, performed more poorly as compared with age-matched TD peers in articulatory and naming skills, lexical comprehension, and lexical knowledge, showing a persisting mild lexical deficit. In addition, their performance on a non-word repetition (NWR) task at t1 correlated with a Semantic Fluency task at t2, showing that the task of NWR, which is an indirect measure of phonological working memory, is also an early indicator of future lexical deficits.

As for grammar abilities, Chilosi et al. [88] assessed 50 Italian-speaking late talkers with measures of receptive and expressive language longitudinally at 28, 36 and 48 months. The authors reported different linguistic outcomes. A first group of children (i.e., “Late Bloomers”) was characterized by a mild expressive delay at 28 months and caught up with their peers at 36 months. A second group (i.e., “Slow Learners”) had mild expressive delay at 28 months that persisted up to 36 months and showed a slow language recovery within 48 months. A third group (i.e., “Children with DLD”) had impaired syntactic comprehension and severe expressive delay at 28 months and difficulties in expressive grammar at 36 months and was diagnosed as DLD at 48 months. These findings suggest that an early syntactic comprehension delay may predict a future diagnosis of DLD in late talkers and confirm the need to include measures of grammar comprehension in late talkers. Nayeb et al. [89] compared the ability of two-word combinations at age 2;5 to that of three-word combinations at age 3 using two language nurse-led screening methods to identify children with DLD. Nurses at three Swedish child health centers observed 105 monolingual Swedish-speaking children at a mean age of 2;5 years. The same children were further assessed by speech and language pathologists at 3 years of age. Both the three-word combination at 3 years and the two-word combination at 2;5 years resulted as accurate measures for the early identification of children with DLD, with, respectively good sensitivity (100% versus 91%), specificity (81% versus 91%), positive predictive (38% versus 56%), and negative predictive value (100% versus 99%). Those children (10%), who were unable to cooperate in the screening at age 2;5, had an increased risk for DLD and should be carefully monitored.

#### 3.2.3. Q1: Summary

In sum, delayed gesture production, limited receptive and/or expressive vocabulary size, impaired syntactic comprehension, and absence of two-word combinations still observable at 30 months of age appear as potential early predictors of DLD. A family history of DLD was often significantly associated with DLD, whereas other biological risk factors such as male gender and pre- and peri-natal conditions were mainly relevant in the first years of life. Associations were also found for low socioeconomic conditions, linguistic input and quality of communicative interactions but with more limited predictive power.

### 3.3. Q2: What Age Range Is Appropriate for the Use of Screening and Diagnostic Tools for DLD?

#### 3.3.1. Results from the Systematic Reviews

Among the systematic reviews, the question is explicitly included only in Nelson et al. [67]. The authors found no studies addressing the question and highlighted the need to derive indications for the identification of optimal ages and intervals for screening from additional work about the effectiveness of school-based interventions.

In their updating of Nelson and collaborators’ review, Wallace et al. [66] confirmed that, although some screening tools can accurately identify language delays or disorders, none of them appear to be more accurate than others, and no age is shown to be particularly adequate for screening. The comparison of the same instrument across different populations showed that some tools (i.e., MB-CDI [113], CSBS ITC [114] and SKOLD [112]) have more robust results than others (i.e., ASQ—Ages and Stages Questionnaire [130]; Fluharty Preschool Speech and Language Screening Test [131,132]). The accuracy of some screening tools seems to drop over time, as observed in two studies on a parent report screening tool administered at 2 and at 3 years of age, which showed lower sensitivity in one study and lower specificity in the other. The authors suggested that the decrease in specificity with time may mean that some children with language delays catch up and display more typical language skills as they grow older [66]. Another reason for the difficulty to identify the optimal age for the appropriate use of diagnostic tools is the need to consider the extreme variability of linguistic and communicative development in children between 3 and 5 years of age.

Albeit not including age among their questions, Law et al. [65] explicitly highlighted the need to explore the relation between age and case definition when discussing the feasibility of universal screenings for speech and language delay. As the authors explicitly pointed out, the issue of early identification requires the previous solution of other issues, namely the need to distinguish between children with speech and language disorders and late talkers in a pre-symptomatic phase (likely in the 1 to 4-year-old age range).

Among the language screening tools examined in the studies reviewed by Sim et al. [69], the best predictive validity performance and diagnostic odds ratio was achieved by the Language Development Survey (LDS) [106,107,108,109], a parent report of vocabulary and word combinations, administered at a mean age of 24.7 months, and the Reynell Developmental Language Scales—RDLS and NRDLS [133,134]—at a mean age of 25.2 months. The second strongest predictive validity data were achieved by the MacArthur Communicative Development Inventory (MB-CDI) Toddler form (ELFRA-2) [115], measuring productive vocabulary, syntax and morphology, administered at an age of 24 months and followed up by the Sprachentwicklungstest für zweijährige Kinder (SETK-3/5) [135], administered at age 37 months. The authors concluded that the age at which children were first assessed does not appear to have a significant effect on the overall predictive performance of the language screening tool used. This conclusion led them to highlight the importance of prioritizing early language skills as a primary child wellbeing indicator and an essential component of routine developmental surveillance in the early years.

Even if they did not directly address the issue, other reviews provided some indirect indications on the appropriate age ranges for the use of diagnostic tools. Maleki Shahmahmood et al. [71] pointed at the preschool period, starting from 3 years, as the most important in the diagnostic process. Kasper et al. [68] did not mention age ranges when presenting their research criteria to assess the potential benefit of systematic population-based screening for DLD in preschool children in Germany. However, in the two publications on screenings included in their review, children’s ages ranged between 15 and 24 months. This suggests that this may be an adequate age range for screenings. Similarly, the data on the 17 speech and language tests analyzed in the diagnostic studies provided a few indications on the age for which they have been designed: some of the tests generally referred to toddlers, kindergarten children, preschoolers or school beginners or even students, while others more specifically mentioned age ranges (precisely 16–26 months, 3 years, 3–5 years, 3–6 years and even 8 years). The conclusion we can derive is that they assume the age between 15 and 24 months as adequate for screenings and that some diagnostic tools for DLD are also used with toddlers for identifying those with a weaker linguistic development. In her review about the factors that may predict preschool-age expressive language outcomes among late talking toddlers, Fisher [72] suggested that the term “late talker” describes “children under age 3 years with unusually small vocabularies and no concomitant developmental disability or hearing impairment”, whereas DLD, which she referred to as Specific Language Impairment (SLI), “is typically diagnosed after age 4”. She included in her review longitudinal studies about toddlers identified as late talkers between 18 and 35 months and who had been administered a follow-up assessment of expressive language before the age of 5 years. Therefore, according to Fisher [72], a timely diagnosis can be performed when the child is between 4 and 5 years old, while children under the age of 3 can only be described as late talkers and, therefore, only at risk for DLD but not yet diagnosed with DLD, without any detail on what happens between 3 and 4 years. Moreover, Fisher [72] highlighted that the relationship between the age at which a child is identified as a late talker and the actual outcome has not been adequately examined yet. Finally, the assessment tools selected by Denman et al. [70] covered the age range from 4 to 12 years. Thus, starting from 4 years onwards is considered appropriate for tests designed to assess language skills across at least two of the domains of spoken language included by the authors.

#### 3.3.2. Results from the Primary Studies

None of the 27 primary studies directly aimed at assessing Q2. Nonetheless, almost all of them highlighted the importance of an early diagnosis and intervention, providing indirect indications about the most appropriate age for the application of diagnostic tools.

First of all, the age groups in the studies aimed at validating questionnaires and tests for the assessment of language and communication development, providing indications on the age ranges considered more appropriate to identify DLD or, at least, potential predictors of DLD. The questionnaires proposed to the children’s parents were found reliable and valid instruments as a first step to assess: (a) motor and language development in the ages ranging from 15 to 38 months in Icelandic toddlers [95]; (b) communication and language development at 24 months in Finnish children [83]; (c) communication abilities in Korean children of an average age of 29;7 months, below 35 months [84]. As far as general tests are concerned, the study on the adaptation, validity and reliability of the Turkish version of the Preschool Language Scale (PLS-5) [97] involved ages ranging from the neonatal stage to 7;11 years. The study [98] concerning the Fluharty-2 test included 3-year-old children but the findings did not confirm its accuracy for this age range. The study concerning the Tamiz de Problemas de Lenguaje—TPL [99]—and the Screening for Identification of Oral—SIOLD [100]—involved 4- to 6-year-old children with diagnostic accuracy increasing toward 5 years of age. In addition, the study by Haman et al. [74], which investigated the cross-linguistic comparability of a tool for the assessment of lexicon in TD children of various languages and cultures, involved ages ranging from 3 to 7;11 years.

Further indications about the ages considered appropriate for the use of diagnostic tools can be derived from the ages of the children included in the studies dedicated to the assessment of their linguistic and communicative abilities to detect potential predictors of DLD. The following age ranges have been identified: (a) use of communicative gestures between 12 and 30 months [75,79,80,81,86]; (b) communication and receptive lexical skills between 15 and 30 months [75,82,83,86]; (c) expressive lexical skills between 15 and 32 months [75,76,82,83,86,87]; (d) syntactic comprehension from 28 months onward [88]; (f) word combination from 30 months onward [89]; (g) non-word repetition from 32 months onward [76]; (h) expressive grammar from 36 months onward [88]. In addition, biological and environmental risk factors for DLD have been detected under the age of 36 months [75,76,77,78,80,81]. Similar indications on the ages at which the non-appearance of certain categories or linguistic skills can be predictive of a DLD can be derived from studies concerning specific aspects of language acquisition in children with TD. For example, Hadley et al. [85] examined the development of communication and expressive vocabulary, including both nouns and verbs, as well as grammar development and emerging grammatical complexity longitudinally, every 3 months, between 21 and 30 months.

Thirdly, the studies on linguistic and communicative abilities in TD children and children already diagnosed with DLD showed that, by the age of 4, a diagnosis has already been made and that, by then, it is possible to use diagnostic tools to collect data about linguistic abilities as well as non-linguistic abilities that can reveal markers associated with DLD. More precisely: (a) 4;6 years is assumed as appropriate to evaluate language skills in general, as well as phonological abilities and precursors of written language [87]; (b) 4 to 5;10 years is the age range to investigate sound discrimination and mapping sound categories to meanings in native English speaking children with DLD compared with age- and gender-matched TD peers [90]; (c) 4 to 6 years is the age range considered appropriate to assess the prosodic skills of Arabic-speaking children with DLD [91]; (d) 4;6 to 5;11 year is the age considered appropriate to investigate the nature of the grammatical deficits of Australian children with DLD [92]; (e) 3;6 to 4;10 years is the age range chosen to evaluate the correlations between linguistic and visual information in word learning in DLD and TD American English children [93]; (f) 5 years is the age to assess both linguistic (articulatory/phonological skills, grammatical production/comprehension, and narrative production) and executive functions (updating and inhibitory tasks) in children with DLD matched with their TD peers [94]; (g) 4 to 6 years in TD children is the age range to assess sentence repetition compared with lexical and grammatical abilities [96].

#### 3.3.3. Q2: Summary

In sum, none of the studies provide explicit indications on the most appropriate age for the use of diagnostic tools. The indirect indications that can be derived are a suggested optimal time for screening between age 2 and 3, whereas diagnosis is optimal around age 4. The appropriate age for the use of single diagnostic tools also varies as a function of the different aspects of language that are measured. As indications about optimal ages for carrying out various assessment procedures have been drawn from research studies, they may also relate to the feasibility of carrying out these procedures with children in these age groups (i.e., the child is able to understand instructions, engage with materials, etc.). In conclusion, as highlighted in many of the selected studies, the issue of the appropriate age for screening and diagnostic tools for DLD is extremely important, as well as complex, and deserves more attention in future research.

### 3.4. Q3: What Tools Are Effective (in Terms of Validity, Accuracy, Reliability) for the Formulation of the Diagnosis of DLD in Preschool Children?

#### 3.4.1. Results from the Systematic Reviews

In order to collect information on the overall psychometric quality of assessment tools and identify those having the best evidence of psychometric quality, Denman et al.’s review [70] systematically examined and evaluated the psychometric quality of diagnostic spoken language assessment tools for children aged 4–12 years using the Consensus Based Standards for the Selection of Health Status Measurement Instruments (COSMIN) checklist [136]. Of the nine measurement properties included in the COSMIN checklist relating to domains of reliability, validity and responsiveness (internal consistency, reliability, measurement error, content validity, structural validity, hypothesis testing, cross-cultural validity, criterion validity and responsiveness), Denman et al. did not consider responsiveness, cross-cultural validity (as all the tools were originally published in English) and criterion validity. Denman et al. [70] took into account tests assessing language skills across at least two of the three domains of semantics, morphosyntax, and discourse. Information was taken from published articles but also from book chapters and manuals. Studies were included if they concerned standardized norm-referenced spoken language assessments, from any English-speaking country with normative data for use with mono-lingual English-speaking children aged 4–12 years. The selected tests included Assessment of Literacy and Language (ALL) [137], the Comprehensive Assessment of Spoken Language (CASL) [138], the Clinical Evaluation of Language Fundamentals for Preschool (CELF:P-2) [139] and for school-aged children (CELF-5) [140], the Diagnostic Evaluation of Language Variance—Norm Referenced (DELV-NR) [141], the Illinois Test of Psycholinguistic Abilities (ITPA-3) [142], the New Reynell Developmental Language Scales (NRDLS) [133,134], the Oral and Written Language Scales (OWLS-2) [143], the Preschool Language Scales (PLS-5) [144], the Test of Early Language Development (TELD-3) [145], the Test of Language Development-Primary (TOLD-P:4) [146], and the Woodcock Johnson Oral Language (WJIVOL) [147]. Based on included studies and manuals, some tests were found to have more psychometric evidence to support their use as diagnostic assessments. These tests include: ALL, CELF-5, CELF:P-2, and PLS-5. The ALL, CELF-5, and PLS-5 were all rated as having “strong” or “moderate” evidence across two or more measurement properties (content validity and hypothesis testing for the three of them, with CELF-5 also having positive ratings for reliability). The CELF:P-2 was identified as having evidence both in content validity and hypothesis testing from the manual; however, information regarding hypothesis testing in the independent literature was conflicting. The ALL, CELF-5, and PLS-5 were not examined in the independent literature. The DELV-NR, ITPA-3, LCT-2, TELD-3, and WJIVOL had only limited evidence for just one measurement property, more precisely for hypothesis testing.

Another review by Maleki Shahmahmood et al. [71] analyzed the literature published in English-language journals between 2000 and 2015, including 23 studies concerning the diagnosis of DLD (in the paper, SLI) in monolingual children of preschool age over the age of 3 years. The included studies were heterogeneous as to the language examined and included: 12 studies with English/American English speaking participants; 3 studies concerning Italian speaking participants; the remaining studies concerned the following languages: Cantonese (*n* = 3), French (*n* = 1), Spanish (*n* = 1), Slovak (*n* = 1), Hebrew (*n* = 1), Persian (*n* = 1). Two types of measures were considered: standardized language tests and a number of psycholinguistic measures extracted from spontaneous language samples.

Overall, the studies included in Maleki Shahmahmood et al. [71] showed sensitivity and specificity values for tests and psycholinguistic measures that varied across the different studies (sensitivity range: 16–100%; specificity range: 14–100%). Among the standardized tests used in the studies on English speaking children, according to Vance and Plante’s [148] criteria for acceptable sensitivity and specificity values, the following results emerged: (a) the “Renfrew bus story”, i.e., a retelling test [149], has adequate sensitivity (84.4) but weak specificity (78.1); (b) grammar production tests have good sensitivity and specificity (i.e., “GAPS” test—Grammatical Additionally, Phonology Screening: sensitivity > 90, specificity > 93 [150,151]; “SPELT-P2” test—Structured Photographic Expressive Language Test—Preschool 2nd edition [152]: sensitivity > 90, specificity > 95; “SPELT-3” test [153]: sensitivity > 90, specificity = 100); (c) vocabulary tests including the Peabody Picture Vocabulary Test PPVT-III [154] (sensitivity = 80, specificity = 75) and the subsequent PPVT-IV version [155] (sensitivity = 80, specificity = 70) have unacceptable levels of sensitivity and specificity that make them inappropriate for identifying children with DLD; (d) as for non-word repetition, the results were not sufficiently consistent across different studies, so it cannot be considered as an accurate diagnostic tool if used individually (e.g., CNRep—children’s test of nonword repetition [156]: sensitivity = 66, specificity = 85; NRT—Nonword Repetition Test [157,158]: sensitivity 79–86, specificity 89–91).

In conclusion, the review by Maleki Shahmahmood et al. [71] suggests that the variability observed in specificity and sensitivity values of the instruments and/or the psycholinguistic measures investigated confirms the need to pay attention to the diagnostic accuracy of each instrument/measure before using it as an effective diagnostic tool in clinical practice. Nevertheless, the two tests measuring grammar/morphosyntactic development, i.e., GAPS [146,147] and SPELT [152,153], showed acceptable psychometric values.

Although the diagnostic power of some psycholinguistic tests/measures seems to be supported by the evidence in the literature, the studies examined by the review have the following limitations: (a) in most cases, the gold standard was represented by experts’ clinical judgment; (b) all the studies were performed on samples composed of an equal number of TD and DLD subjects (prevalence 50%) and most of them on unselected populations; (c) children with DLD were recruited from those under treatment; (d) variability and arbitrariness in the choice of the cut-off of the index test have significantly influenced the results in terms of accuracy values. The review highlighted the importance of investigating the joint use of multiple measures to increase diagnostic accuracy.

#### 3.4.2. Results from Primary Studies

One study [97] aimed to verify cross-cultural adaptation and to assess the validity and reproducibility of the Turkish version of the Preschool Scale for Language (TPLS-5) [144]. The PLS-5 is an English test that also has a French version and is used for the assessment of both receptive and expressive language. The study was conducted on a large sample of participants (1320 children, including 276 with receptive and/or expressive language disorder) with a wide age range (0–7 years and 11 months). However, its subdivision into numerous age groups produced sub-samples that are not representative enough for each of the sub-groups. The study showed the good validity of TPLS-5 in the identification of language disorders in Turkish children. Benavides et al. (2018) [99] assessed the concurrent validity of the morphology cloze task and the sentence repetition task of the Tamiz de Problemas de Lenguaje (TPL), for identifying 4- to 6-year-old monolingual Spanish speaking children at risk of DLD with grammatical deficits. The sample consisted of 770 children, including 586 children with TD and 184 participants with DLD, divided into three age groups. The TPL screening task showed good sensitivity and fair specificity for 4- (0.90 and 0.83, respectively) and 5-year-olds (0.90 and 0.84, respectively) and good sensitivity and specificity for 6-year-olds (0.94 and 0.92, respectively), with positive and negative likelihood ratios being moderate to large. The authors concluded that the TPL has high accuracy in identifying children at risk for DLD with grammatical deficits. Puglisi et al. [100] developed and validated the Screening for Identification of Oral Language Difficulties by Preschool Teachers (SIOLD), a screening questionnaire on phonology, vocabulary, and grammar for the early identification of language difficulties in Brazilian Portuguese-speaking children aged 4- to 6-years-old. Its accuracy was tested on 100 children ranging from 5;00 to 6;08 years. The SIOLD showed acceptable sensitivity (ranging between 0.75 and 0.86) and good specificity (0.95) values. Although the SIOLD overpredicted positive cases, it identified most children with true language disorders and passed most children without language disorders. Thus, it proved useful for Brazilian preschool teachers to refer children with language difficulties between 5 and 6 years of age to the speech language services. Further research is, however, needed on larger samples also including children younger than 5. Finally, Eisenberg et al. [98] examined the concurrent validity of the Fluharty-2 test [132] by comparing its performance in 62 3-year-old children (31 who had failed the test, and 31 who had passed it) to four diagnostic measures: the SPELT-P2 [152]; mean length of utterance in morphemes (MLUm), a finite verb morphology composite, and the Index of Productive Syntax (IPSyn). Children who failed the Fluharty-2 scored significantly lower on each of the diagnostic measures than children who passed the Fluharty-2, but the effect size for MLUm was small. The authors concluded that the Fluharty-2 would refer too few children at risk and too many non-at-risk children for a follow-up assessment, making it an inefficient tool for mass screenings of language at 3 years of age.

#### 3.4.3. Q3: Summary

In sum, the selected literature did not provide sufficient data to identify effective single tools for diagnosis of DLD in preschool children before 5 years of age. A joint use of standardized and psycholinguistic measures such as grammatical markers (e.g., subject/verb agreement, gender, tense and number inflections, free morphemes, clitics, etc.), or production measures (e.g., Mean Length of Utterance—MLU) is suggested to increase diagnostic accuracy, with grammatical tests generally showing increasing accuracy in identifying children with DLD from 5 years onwards.

## 4. Discussion

By employing an evidence-based approach, the current scoping review aimed at identifying (a) early predictors of DLD; (b) the most appropriate age range for the use of screening and diagnostic tools; (c) reliable diagnostic tools of DLD with good psychometric properties in preschool children. We have not always found direct answers, but the analysis of the literature has highlighted relevant findings.

Delayed gesture production, limited receptive and/or expressive vocabulary size, impaired syntactic comprehension, and the absence of two-word combinations up to 30 months of age emerged as potential early predictors of DLD. A family history of DLD was found often significantly associated with DLD, whereas other biological risk factors such as male gender and pre- and peri-natal conditions were mainly relevant in the first years of life. Even if with a lower predictive power, associations were also found for low socioeconomic status, linguistic input, and quality of communicative interactions. The available literature suggests that a timely screening can be performed between 2 and 3 years of age, whereas a timely DLD diagnosis can be performed around 4 years of age. Because sensitivity and specificity values of diagnostic tools and psycholinguistic measures show high variability, a joint use of standardized and psycholinguistic measures (e.g., the analysis of the use of bound or free morphemes or a narrative sample’s MLU) is suggested to increase diagnostic accuracy. Such accuracy increases around 5 years of age, especially for grammatical tests. These and other relevant issues are discussed in the following sections of the Discussion.

### 4.1. Risk Factors and Early Predictors

The first issue concerns the distinction between risk factors and early predictors. These terms refer to two different types of indicators, whose distinct characteristics encourage treating them separately. Unfortunately, this has not always been performed in published studies. Treating these factors separately can increase the accuracy of predictions. Among risk factors, biological ones are generally more predictive in the very first years of life, whereas psychosocial risk factors become increasingly more important during development [73,159]. As also suggested by Bettio et al. [73], risk factors interact with each other in complex ways. Unfortunately, the different studies have not devoted the same attention to all kinds of risk factors. Indeed, there is a prevalence of studies on biological factors while just a few studies have focused on environmental risk factors, dynamic risk factors (such as family interactions) and protective factors. It can thus be concluded that research taking into account several types of factors at the same time and characterizing their interactions should be encouraged.

Early predictors, on the other hand, are observable, individual traits or behaviors that predict future language development. For example, these might include the use of gestures, vocabulary size (both receptive and expressive), and early grammatical skills. Here, we would like to highlight the difficulty of clearly distinguishing between predictors of general language development and predictors of DLD. Clearly, these belong to a continuum, although it is essential to focus on the latter more than on the former. The observed difficulty may be, at least in part, related to the heterogeneity of nosographic categories and labels used in the literature. It may stem from the heterogeneity of diagnostic criteria and cut-offs across different studies as well. Obviously, this may lead to the inclusion of different clinical populations and different conclusions (see also [22]). Noteworthy, we should also highlight that the predictive power of some of the reported indicators changes over time and that some of the predictors reported in the selected studies referred to late talkers, while others referred to the prediction of fully diagnosed DLD. Additionally, some of the investigations on the predictors of persistent language delay were general population studies (e.g., [75]), while others referred to late talkers (e.g., [72,86,88]) or children at risk of language delay (e.g., [79,80,81]).

### 4.2. Screening Tools and Early Predictors

The second issue regards the distinction between screening tools and early predictors. Screening tools often include early predictors among their items. Unfortunately, such predictors have almost never been analyzed in terms of their specific, individual predictive and discriminating power, even in studies where such indicators have been provided for the whole screening tool. For these reasons, we decided to include studies concerning screenings, even though they may not include any information about single early predictors of the disorder. It should also be considered that different screening tools exist, including or not including testing of children along with parental (or clinicians’) questionnaires. For these reasons, screening as a tool to identify children at risk for DLD has been treated separately from single early predictors.

Beyond general support for the validity of screening tools at an early age, little evidence was found for the usefulness of screening procedures in order to reduce the incidence of the disorders: even when the screenings correctly identified children at risk, evidence regarding the efficacy of an intervention aimed at modifying the children’s developmental trajectories is scarce. Therefore, further research on the efficacy of routine screening for the identification of preschoolers at risk of DLD is needed. In our opinion, such studies should employ multifactorial models that take into account both risk factors and early predictors of a poor language prognosis with repeated measurements over time, as suggested by Nelson et al. [67] and also attempted by one of the studies reviewed in Bettio’s study [73], albeit with negative results.

### 4.3. Timely Identification

A third issue concerns age and the importance of identifying DLD within the preschool period, by identifying children at risk within the age of 3 years and by making a diagnosis between 4 and 5 years, whereas the status of children between 3 and 4 years remains unspecified. The importance of an early detection is widely recognized, but the identification of a more precise optimal age range is not easy for a number of reasons: (a) the difficulty to distinguish between delay and disorder and to identify a pre-symptomatic phase that may differentiate between them; (b) the extreme variability of linguistic and communicative development in children between 3 and 5 years; (c) the need to take into account the variability of the age of emergence of the different language skills so that, at a certain age, it is possible to assess some linguistic levels and not others. The assessment should also be extended to non-linguistic abilities (e.g., sound discrimination, expressive prosody, phonological working memory, associative learning, executive skills) that may significantly contribute to children’s linguistic difficulties [90,91,92,93,94].

### 4.4. Diagnostic Tools

The revised literature provides limited evidence on the diagnostic accuracy of the tools used. As suggested by Maleki Shahmahmood et al. [71], in order to validate their use in clinical practice, it would be essential to collect data from: (a) replication studies for the same measures in independent samples; (b) studies using different measures in the same sample; (c) cross-linguistic studies, in particular for the assessment of nominal morphology, verbal morphology or other grammatical aspects that vary greatly among languages.

## 5. Limitations and Future Perspectives

The present review has some limitations. A first limitation, emerging especially from the systematic reviews, concerns the generalizability of the results and paucity of data on languages other than English. Some of the early predictors (i.e., communicative gestures [75,79,80,81,86]; communicative skills [75,82,83,86]; receptive and productive vocabulary size [67,73,75,76,82,83,85,86,87]; nonverbal cognitive abilities [73]) can be easily observed regardless of the language of the children. Unfortunately, the same cannot be said for psycholinguistic measures. On the one hand, the paucity of cross-linguistic studies does not facilitate the identification of potentially universal diagnostic markers of DLD among different language-specific psycholinguistic measures. On the other hand, the psychometric characteristics of diagnostic tools can hardly be generalized across different languages, due to the differences between languages at all levels (e.g., phonological, morphological, syntactic, etc.) and to the variability in their emergence during development. Nonetheless, some features can be generalized across language families. Furthermore, some factors apparently cross language boundaries and are always present, even if with different impacts according to the characteristics of the specific language. According to Leonard and Kueser [160], among such factors we might include: (1) the status of bare stems in the language; (2) the use of grammatical case; (3) the role of prosody; (4) interactions between aspect and tense; and (5) the canonical word order of the language. Indeed, some studies providing data on languages other than English do exist. For instance, there are interesting studies on DLD screening and diagnostic tools in Romance languages, but they are mainly published in national reviews or are based on relatively small samples; therefore, they did not satisfy selection criteria and have not been included in the present review.

This contributes to a further limitation, namely the difficulty to translate the results into recommendations for clinical services. As for early screenings, due to the lack of clear indications on their efficacy to reduce later incidence of DLD, it is not easy to recommend a generalized use of these practices. Nonetheless, it has to be considered that poor efficacy seems to depend on the difficulty to significantly influence and modify the developmental trajectories of children at risk for DLD through intervention, rather than to the poor capacity of the tools to identify children at risk (sensitivity and specificity features of several tests have proved to be good). This consideration should therefore encourage research on effective intervention practices in particular [33], and suggests that a good association between the use of reliable screening tools and targeted interventions could be the desired solution.

Similarly, as noted also by Denman et al. [70], the limited evidence on the diagnostic accuracy of the tools used in clinical practice should not be taken as evidence of low levels of accuracy and reliability, but rather as evidence of the need for dedicated studies.

## 6. Implications for Clinical Practice

In spite of such limitations, some recommendations for clinical practice can be derived from the present review, which we would like to outline in the conclusive paragraphs.

The path that leads to the diagnosis of DLD typically involves: (a) first, identifying children at risk of developing persistent language deficits with the use of reliable screening tools; (b) at a later time, if language deficits persist, assessing their type and severity to ascertain whether a diagnosis of DLD is needed; (c) finally, for treatment planning, assessing environmental context, available resources, needs and priorities responding to the needs and expectations of children and families.

As for the timing at which risk for DLD should be screened and DLD assessed, studies have focused on children aged 5 years and younger; however, unclear results have been reported about the optimal age, frequency and efficacy of screening. Screening a large set of children, including children with low risk, increases the probability of false positives. However, delayed identification has long-lasting negative consequences.

Relatives, teachers or healthcare professionals could be highly accurate observers to note early predictors or concomitant markers of the presence of DLD. The collected evidence suggests the opportunity to exploit the potential contribution from people outside the healthcare system but with a deep knowledge of the child. If properly trained and supervised by experts, they could complete screening questionnaires and systematically observe/record the behaviors that could suggest the presence of DLD (for example, gesture and lexical production).

Clearly, the use of reliable screening and diagnostic tools presupposes the existence of shared scientific literature providing all the relevant information, such as sensitivity, specificity, replicability, and population representativeness, involved in tool validation.

## 7. Conclusions

Monitoring risk situations and employing caregivers’ reports, clinical assessment and multiple linguistic measures are fundamental for an early identification of DLD and the planning of timely interventions. Further research is needed to collect such data, possibly also including children who are bi- and multilingual, especially in languages and cultures other than English, to improve the predictive value of diagnostic tools and ensure correct and early identification of DLD in preschool children.

## Data Availability

Not applicable.

## Appendix A

**Table A1:** Search strategy for PubMed.

	PubMed Date: 31 December 2020
Search	Query
#15	#13 AND #14
#14	systematic[sb] OR meta-analysis[pt] OR meta-analysis as topic[mh] OR meta-analysis[mh] OR meta analy*[tw] OR metanaly*[tw] OR metaanaly*[tw] OR met analy*[tw] OR integrative research[tiab] OR integrative review*[tiab] OR integrative overview*[tiab] OR research integration*[tiab] OR research overview*[tiab] OR collaborative review*[tiab] OR collaborative overview*[tiab] OR systematic review*[tiab] OR technology assessment*[tiab] OR technology overview*[tiab] OR “Technology Assessment, Biomedical”[mh] OR HTA[tiab] OR HTAs[tiab] OR comparative efficacy[tiab] OR comparative effectiveness[tiab] OR outcomes research[tiab] OR indirect comparison*[tiab] OR ((indirect treatment[tiab] OR mixed-treatment[tiab]) AND comparison*[tiab]) OR Embase*[tiab] OR Cinahl*[tiab] OR systematic overview*[tiab] OR methodological overview*[tiab] OR methodologic overview*[tiab] OR methodological review*[tiab] OR methodologic review*[tiab] OR quantitative review*[tiab] OR quantitative overview*[tiab] OR quantitative synthes*[tiab] OR pooled analy*[tiab] OR Cochrane[tiab] OR Medline[tiab] OR Pubmed[tiab] OR Medlars[tiab] OR handsearch*[tiab] OR hand search*[tiab] OR meta-regression*[tiab] OR metaregression*[tiab] OR data synthes*[tiab] OR data extraction[tiab] OR data abstraction*[tiab] OR mantel haenszel[tiab] OR peto[tiab] OR der-simonian[tiab] OR dersimonian[tiab] OR fixed effect*[tiab]
#13	#1 AND #10 AND #11 AND #12
#12	Child[Mesh] OR Infant[Mesh] OR child*[tiab] OR infant*[tiab] OR baby[tiab] OR babies[tiab] OR toddler*[tiab] OR boy*[tiab] OR girl*[tiab] OR pre-school*[tiab] OR preschool*[tiab] OR kindergarten*[tiab] OR kinder-garten[tiab] OR nursery[tiab]
#11	test*[tiab] OR instrument[tiab] OR judgments[tiab] OR scale[tiab] OR tool*[tiab] OR procedure*[tiab] OR assessment [tiab] OR assessing[tiab] OR vignette*[tiab] OR scenario*[tiab] OR “rating scale”[tiab] OR “rating scales”[tiab] OR “coding manuals”[tiab] OR “coding schemes”[tiab] OR checklist*[tiab] OR interview*[tiab] OR questionnaire*[tiab]
#10	#2 OR #3 OR #4 OR #5 OR #6 OR #7 OR #8 OR #9
#9	reliability[tiab]
#8	early identification [tiab]
#7	accuracy[tiab]
#6	“Sensitivity and Specificity”[Mesh]
#5	“Predictive Value of Tests”[Mesh]
#4	“reproducibility of Results” [MESH]
#3	Diagnosis”[Mesh] OR “diagnosis” [Subheading]
#2	diagnosis”[tiab] OR “diagnostic”[tiab]
#1	“Language Disorders”[Mesh] OR “Speech Sound Disorder”[Mesh] OR speech disorder*[tiab] OR speech delay*[tiab] OR speech impair*[tiab] OR language disorder*[tiab] OR language delay*[tiab] OR language impair*[tiab] OR language difficulties[tiab] OR phonological disorder* [tiab]

## Appendix B

**Table A2:** Characteristics of the 10 selected systematic reviews.

Authors, Year, Reference	Goal of the Review	Study Design (*n*° of Studies)	Population	Language of Assessment	Language Domain/Task	Test/Assessment Tool
Bettio et al., 2019 [73] Data search: 2013–2017	Identify: (a) risk factors associated with delays in the development of children’s oral language (b) protective factors that could moderate the effects of risk factors associated with oral language delays	Systematic reviews (*n* = 2), cohort studies, longitudinal (*n* = 8), cross-sectional studies (*n* = 2).	Size of total sample not specified Age = Birth to 8 years	Not specified. Countries where studies were conducted: Finland (1), Ireland (1), Brazil (3), Canada (2), Australia (2), Norway (1), Scotland (1) and USA (1)	Receptive–expressive language (language development in general)	(I) Static risk factors: Male gender, low birth weight, preterm birth, low parental schooling, low socioeconomic status, ≥4 children living in the same household, family history of language delay, father working outside all day, difficult temperament, intracranial hemorrhage, brain injury and persistent otitis media. (II) Dynamic risk factors: Poor quality of communication with the mother, family dynamics, family not reading to the child at home, and problems with the mother’s mental health.
Denman et al., 2017 [70] Data search: 1994–2014	Evaluation of psychometric quality of diagnostic spoken language tests for monolingual English-speaking children	Manuals of tests (*n* = 12), diagnostic accuracy studies (*n* = 7)	Size of total sample not specified Age: range 4–12 years	English	Spoken and written language skills including phonemic awareness and pragmatics	Tests assessing language skills across at least two domains of word (semantics), sentence (syntax/morphology) and text (discourse): Assessment of Literacy and Language [ALL]; Comprehensive Assessment of Spoken Language [CASL]; Clinical Evaluation of Language Fundamentals—5th Edition [CELF-5], Clinical Evaluation of Language Fundamentals: Preschool—2nd Edition [CELF:P-2], Diagnostic Evaluation of Language Variance—Norm Referenced [DELV-NR], Illinois Test of Psycholinguistic Abilities—3rd Edition [ITPA-3], Reynell Developmental Language Scales—4th Edition [NRDLS], Oral and Written Language Scales—2nd Edition [OWLS-2], Preschool Language Scales—5th Edition [PLS-5], Test of Early Language Development—3rd Edition [TELD-3], Test of Language Development—Primary: 4th Edition [TOLD-P:4], Woodcock Johnson 4th Edition Oral Language [WJIVOL]
Fisher, 2017 [72] Data search: until July 2015	Analysis of predictors of expressive language outcomes among late talkers	Prospective studies (*n* = 23), 1 dissertation, 11 personal communications, corresponding to 20 LT samples.	N = 2134 59% boys, 41% girls Age: 18–35 months, with 5 months follow-up assessment	American English, British English, Dutch, Australian English, Finnish, Greek, French, Serbian	Expressive language	Predictors *Continuous:* expressive vocabulary size, receptive language, phrase speech, socioeconomic status *Nominal:* gender, family history.
Kasper et al., 2011 [68] June–October 2007, updated January and May 2008.	Evaluation of the effectiveness of a screening program, diagnosis and interventions for specific language impairment (SLI).	Screening: Cluster randomized controlled studies (*n* = 2)	Total sample: N = 10,942 15–24 months with 2 years follow-up period	German	Spontaneous language production (expressive lexicon and morphosyntax)	VTO language screening
Law et al., 1998 [64] Data search: 1966–1997	Evaluation of screening procedures for speech and language delays.	Cross-sectional studies (*n* = 45)	0–7 years	English	Receptive–expressive language Articulation	One screen/multiple populations: Fluharty Preschool Language Screening test, Sentence Repetition Screening Test, Northwestern Syntax Screening test, Revised Denver Developmental Screening Test Expressive/Receptive (DDST), Battelle Developmental Inventory Screening Test, Parent Questionnaire with/without comprehension items, Nurses Developmental Screening, Speech and language Screening Questionnaire; WILSTAAR, SKOLD, ELM, Hackney, LDS, Levett-Muir, Rigby Speech Screen, Stevenson Screen, TPSI, Uppsala Language Screen.
Law et al., 2000 [65] Data search: 1967–May 1997	Evaluation of the feasibility of universal screening for speech and language delay	Cross-sectional studies (*n* = 45)	Sample size not specified Age: 5–70 months	British and American English	Receptive–expressive language	1. Single screening applied to more than one population: Fluharty Preschool Language Screening Test and Sentence Repetition Screening Test versus Test of Language Development and Test of Auditory Comprehension of Language. 2. Comparison of more than one screening applied to a single population: Fluharty Preschool Language Screening Test, Northwestern Syntax Screening test, Revised Denver developmental Screening Test Expressive/Receptive, Battelle Developmental Inventory Screening Test, Parent Questionnaire with/without comprehension items, Nurses Developmental Screening, Sentence Repetition Screening Test, Speech and Language Screening Questionnaire
Maleki Shahmahmood et al., 2016 [71] Data search: 2000–July 2015	Evaluation of accuracy of language tests/measures for the diagnosis of Speech and Language Impairment. Evaluation of the possibility to identify universal linguistic markers of DLD.	Cross-sectional studies that compare the performance of two or more diagnostic procedures (*n* = 23)	Total sample: N = 2784 (range 29–454) Preschool age (from 36 months)	12 studies on English or American English-speaking children, 11 studies on children speaking other languages: Cantonese (3), Italian (3), French (1), Spanish (1), Slovakian (1), Hebrew (1), Persian (1)	-Receptive–expressive language	Studies in English: non-word repetition, experimental test and digit task, language tasks, CNRep, Spelt-P3, NRT, Bus Story, SPELT-P2, GAPS-test, TMT, PS, FVMS, PPVT-III, PPVT-IV, spontaneous language; reference test: clinical assessment by professionals, other tests
Nelson et al., 2006 [67] Data search: 1966–Nov. 2004	Evaluation of screening and interventions for speech and language delay in primary care setting	Case control, cross-sectional, prospective cohort studies, (*n* = 38)	KQ2a: N = 13,787; KQ2b and 2c: N = 1627. Total sample N = 15,414 CA < 5 years	American English, German, Dutch, Finnish	-Receptive–expressive language	0–2 years: Early Language Milestone Scale (2), PEDS (1), DDST-II (1), PLASTER (1), CLAMS (1), LDS (3), DP-II (1), BINS(1); 2–3 years: PLC (1), Structured Screening Test (1), Levett–Muir Language Screening Test (1), Fluharty Preschool Speech and Language Screening Test (2), SKOLD (1), Hackney Early Language Screening Test (2), Early Language Milestone Scale (1); 3–5 years: Fluharty Preschool Speech and Language Screening Test (1), TEEM (1), SRST (1)
Sim et al., 2019 [69] Data search: Medline 1946–March 2017, Embase 1947–2017, EBSCO CINAHL 1983–2017, PsycInfo 1914–2017 and ERIC 1959–2017	Evaluation of the predictive validity of screening tools for language difficulties used in a community preschool setting	Prospective cohort studies (*n* = 5)	Total sample = 9267 Age: 2–6 years	English (N = 2), German (N = 1), not specified for the remaining studies (probably English as they use English tests)	-Receptive–expressive language -General cognitive development	MB-CDI: UK Short Form (MB-CDI: UKSF) or Toddler form (ELFRA-2); Parent Report of Children’s Abilities (PARCA); Language Development Survey (LDS); Reynell Developmental Language Scales (RDLS); Sprachentwicklungs test (for 2-year-olds) SETK-2; nonverbal subscale of the Munchener Funktionelle Entwicklungsdiagnostik, hearing screen ECHO-SCREEN Plus-T; Productive vocabulary, syntax and morphology; Parent reports; Sure Start Language Measure (SSLM); Strengths and Difficulties Questionnaire (SDQ); Vocabulary; Development and Wellbeing Assessment (DAWBA); Griffiths Mental Development Scale-Extended Revised (GMDS-ER); New Reynell Developmental Language Scales (NRDLS); General Language Screen (GLS); Developmental Profile II (DPII); Receptive and expressive language.
Wallace et al., 2015 [66] Data search: Jan. 2004–July 2014	Evaluation of efficacy of screening and treatment for speech and language delays and disorders (update of Nelson, 2006)	Longitudinal studies (*n* = 24) (KQ1: 0 studies; KQ2a: 24 studies; KQ2b, KQ2c, KQ2d, KQ3, KQ4: 0 studies).	Total sample: 7823 7–72 months (7–54 screening by parents, 18–72 screening by clinicians)	KQ2a: American English, German, Swedish, Spanish	-Receptive–expressive language -Gestures -Sounds -Object use	KQ2A: Tools compiled by parents: PLS-3 o PLS-4; language observation; ELFRA-2 words and sentences (SETK-2); DP II, EAT, RDLS, BPVS; language inventory RAPT; toddlers Inventory CSBS; clinical assessment on MSEL; MLU, RDLS; parent questionnaires; SLP on language samples; REEL word screening; Tools used by clinicians: Battelle Developmental Inventory Screening test; Brigance Screening; Davis Observation Checklist; Denver test of articulation; Denver screening; Early Profile of verbal concepts PLS-4; Fluharty Preschool Language Screening test; FPSLST Language and Articulation; Northwestern; SKOLD; sentence repetition; structured screening test; Hackney, RDLS; SLP.

**Table A3:** Characteristics of the 27 selected primary studies.

Authors, Year, Reference	Study Design, Setting	Participants	Age (Months)	Language of Assessment	Language Domain/Task	Test/Assessment Tool
Azab and Ashour, 2015 [91]	Observational, cross-sectional; clinical setting	N = 60 (30 DLD + 30 TD)	48–70	Arabic	-Prosody	-Protocol of prosodic assessment; Clinical criteria: -Arabic language test
Benavides et al., 2018 [99]	Observational, cross-sectional; clinical setting	N = 770 (184 DLD + 586 TD) 4 y = 73 DLD + 189 TD 5 y = 63 DLD + 245 TD 6 y = 48 DLD + 152 TD	48–72	Spanish (Mexico)	-Expressive grammar	Tamiz de Problemas de Lenguaje (Morphology task and sentence repetition task) Clinical criteria: -Subtests of BESA = Bilingual English–-Spanish Assessment. -CELF-4 = Clinical Evaluation of Language Fundamentals -MLU = mean length of utterance
Bello et al., 2018 [86]	Observational, longitudinal; clinical setting	N = 35 LT	T0: 29 T1: 34	Italian	- Receptive and expressive lexicon -Socio-conversational abilities	Clinical criteria: -MacArthur–Bates Infant and Toddler Communication Development Inventories. (Italian version of the MB-CDI Short and Long Form) -Parole in Gioco-PiNG, Test of noun and predicates comprehension and production -Questionnaire of socio-conversational abilities- ASCB
Chilosi et al., 2019 [88]	Observational, longitudinal; clinical setting	N = 50 LT	T0:28 T1:36 T2:48	Italian	-Expressive lexicon -Expressive grammar -Syntactic comprehension	Clinical criteria: -MacArthur–Bates Infant and Toddler Communication Development Inventories. (Italian version of the MB-CDI) -Grid for the Analysis of Spontaneous Speech (GASS). -COVER test of syntactic comprehension
Collisson et al., 2015 [93]	Experimental, cross-sectional; clinical setting	N = 54 (16 DLD + 38 TD)	42–58	English	-Receptive and expressive lexicon -Expressive grammar	Clinical criteria: -CELF-P2 = Clinical Evaluation of Language Fundamentals -Preschool 2 -PPVT-4 = The Peabody Picture Vocabulary Test—Fourth Edition
Conway et al., 2018 [78]	Observational, longitudinal; home setting	N = 197 former LT	T0 24 T1:36 T2:48	Australian English	-Receptive and expressive lexicon -Receptive and expressive grammar	Maternal speech in mother–infant interaction Clinical criteria: -The Preschool Language Scale (PLS-4) -The Clinical Evaluation of Language Fundamentals Preschool Edition (CELF-P2)
Eisenberg et al., 2019 [98]	Observational, cross-sectional; setting non specified	N = 62 (31 LT and 31 TD)	36	American English	-Expressive lexicon -Expressive grammar	-Fluharty Preschool Speech and Language Screening Test–2ed Clinical criteria: -SPELT-P2 = Structured Photographic Expressive Language Test–Preschool, Second Edition. -MLUm = mean length of utterance in morphemes. -IPSyn = Index of Productive Syntax. -FVMC = finite verb morphology composite.
Gudmundsson, 2015 [95]	Observational, cross-sectional; home + clinical setting	N = 1132 (general population)	15–38	Icelandic	-Receptive and expressive language	Toddler Language and Motor Questionnaire
Hadley et al., 2016 [85]	Observational, longitudinal; clinical setting	N = 45 TD	T0: 21 T1: 24 T2: 27 T3: 30	American English	-Expressive lexicon -Expressive grammar	-Word and sentences version of MB-CDI (21, 24, 27, 30 months). -Recordings of spontaneous language (21–30 months) obtaining measures of expressive language abilities: total length of complete and interpretable utterance, number of word types, mean length of utterance in morphemes (24 and 30 months), measures of vocabulary as number of different nouns and verbs
Haman et al., 2017 [74]	Observational, cross-sectional; school setting	N = 639 TD	36–82	Lithuanian, isiXhosa, Finnish, Afrikaans, British English, South African English, German, Luxembourgish, Norwegian, Swedish, Catalan, Italian, Hebrew, Polish, Serbian, Slovak and Turkish	-Expressive lexicon	Cross-linguistic lexical tasks (LITMUS-CLT)
Hsu and Iyer, 2016 [75]	Observational, longitudinal; setting not specified	N = 1064 general population	T0: 15 T1: 36 T2: 53	American English	-Gesture production -Receptive and expressive lexicon	-MacArthur–Bates Communicative Development Inventory (MB-CDI) Clinical criteria: -Reynell Development Language Scales (RDLS, 3 years) -Preschool language scale-3 (PLS-3, 4.5 years)
Kademann et al., 2015 [87]	Observational, longitudinal; clinical setting	N = 86 (46 LT + 40 TD)	T0: 24 T1: 36 T2: 54	German	-Receptive and expressive lexicon -Expressive grammar -Phonology - Narrative production -Metaphonology -Verbal memory -Lexical access -RAN (rapid automatized naming)	Clinical criteria: -SETK-4-5: sentence comprehension (VS), morphological production (plural formation—MR), sentence memory (SG) -Subtest Vocabulary of K-ABC battery
Kim et al., 2016 [84]	Observational, longitudinal; clinical setting	N = 206 with delayed language development (79 DLD + 19 TD + other pathologies)	29.7 (average age)	Korean	-Receptive and expressive lexicon -Receptive and expressive grammar	-Korean version of Ages and Stages Questionnaire (K-ASQ), -MacArthur–Bates Communicative Development Inventories short Korean version (MB- CDI-K) Clinical criteria: -SELSI (Sequenced Language Scale for Infants), -PRES (Preschool Receptive–expressive Language Scale)
Klem et al., 2015 [96]	Observational, longitudinal; school setting	216 TD monolingual children	T0: 51 T1: 63 T2: 75	Norwegian	-Sentence repetition -Expressive grammar	-Sentence repetition test -‘Grammatic Closure’ from Illinois Test of Psycholinguistic Abilities (ITPA)
Lüke et al., 2017 [79]	Experimental, longitudinal; clinical setting	N = 59 TD Recruitment strategy emphasized families in which a sibling or one of the parents had a history of language impairment.	T0: 12 T1: 24	German	-Gesture comprehension and production -Receptive and expressive lexicon -Receptive and expressive grammar	-Analysis of gestural behavior (pointing) at 1;0 year in a semi-natural setting with their caregivers Clinical criteria: -SETK-2 -FRA-KIS
Lüke et al., 2020 [80]	Observational, longitudinal (14 sessions from 1 to 6 years); clinical setting	N = 42 children (TD: N = 32; LD: N = 10 (at 24 months); of children with LD, N = 2 with DLD from age 3. Same recruitment strategy as in [79].	T0: 1;0; T1: 1;2; T2: 1;4; T3: 1;6; T4: 1;9; T5: 2;0; T6: 2;6; T7: 3;0; T8: 3;6; T9: 4;0; T10: 4;6; T11: 5;0; T12: 5;6; T13: 6;0.	German	-Gesture production and comprehension -Receptive and expressive lexicon -Receptive and expressive grammar -Sentence repetition	-Analysis of gestural behavior (pointing) at 1;0 year in a semi-natural setting with their caregivers -Iconic gesture test to assess comprehension of iconic gestures at 3;0, 4;0 and 5;0 years Clinical criteria: -German version of the MB-CDI (at 24 months) -ELFRA (at 12 months) -FRAKIS and SETK-2 (at 24 and 30 months) -SETK 3-5 and PDSS (at 36 months) -P-ITPA (at 48, 60, 72 months) -TROG-D (at 60, 72 months) and receptive language
Marini et al., 2017 [76]	Observational, longitudinal; clinical setting	N = 293 (260 TLD + 33 LT)	T0: 32 T1: 41	Italian	-Non-word repetition -Expressive lexicon -Phonology/Articulation -Receptive and expressive grammar -Narrative production	-Non-word repetition -Language Development Survey (LDS) -Home Literacy Environment Questionnaire (HLEQ) Clinical criteria: -Batteria per la Valutazione del Linguaggio in Bambini dai 4 ai 12 anni (BVL_4-12)
Marini et al., 2020 [94]	Observational, cross-sectional; clinical setting	N = 40 (TD: N = 24; DLD: N = 16)	64	Italian	-Phonology/Articulation -Receptive and expressive grammar -Narrative production	Inhibition test (NEPSY-II) Clinical criteria: -Batteria per la Valutazione del Linguaggio in Bambini dai 4 ai 12 anni (BVL_4-12)
Morgan et al., 2020 [82]	Observational, longitudinal; clinical/home setting	N = 408 (159 LT + 249 TD)	T0: 18–21 T1: 24 T2: 36	American English	-Communication and symbolic abilities -Receptive and expressive lexicon	-Communication and Symbolic Behavior Scales (CSBS) -Language Development Survey (LDS) Clinical criteria: -Mullen Scales of Early Learning (MSEL)
Nayeb et al., 2019 [89]	Observational, longitudinal; clinical setting	N = 105 (11 DLD + 94 TD)	T0: 30 T1: 36	Swedish	-Word combination	-Evaluation of word combination in child speech -Comprehension test; Clinical criteria: Reynell Development Language Scales- RDLS-III (Swedish); Evaluation of spontaneous communication
Puglisi et al., 2020 [100]	Observational, cross-sectional; school setting	Study 1: 754 TD Study 2: 100 (92 TD e 8 LD)	Study 1: 51–65 Study 2: 60–80	Brazilian-Portuguese	-Phonology -Expressive lexicon -Expressive grammar	-Screening for Identification of Oral Language Difficulties by Preschool Teachers (SIOLD) (questionnaire on phonology, vocabulary, grammar). Clinical criteria: -Expressive Vocabulary Test- Short -Test for Reception of Grammar Version 2—Short -The Brazilian Children’s Test of Pseudoword Repetition
Quam et al., 2020 [90]	Observational, cross-sectional; school setting	N = 52 (26 DLD + 26 TD)	48–70	American English	-Sound discrimination -Receptive and Expressive Lexicon	-Computerized sound discrimination task Clinical criterion: -Peabody Picture Vocabulary Test, Fourth Edition (PPVT-4). -Structured Photographic Expressive Language Test—Preschool: 2nd Edition (SPELT-P2)
Sahli and Belgin, 2017 [97]	Observational, cross-sectional; clinical setting	N = 1320 (1044 TD; 276 DLD)	0–95	Turkish, English	-Auditory Comprehension -Expressive Communication	Clinical criteria: -Preschool language Scale 5 ed. (PLS-5) -Preschool language Scale 4 ed. (PLS-4)
Sansavini et al., 2019 [81]	Observational, longitudinal; Clinical setting	N = 110 infants (70 American: -29 TD −41 siblings with no ASD of a child with ASD: 28 no LD, 13 LD; 40 Italian infants: −20 Full Term −20 Extremely preterm: 11 no LD, 9 LD)	T0: 18 T1: 24 T2: 30 T3: 36	American English Italian	-Gesture production -Expressive lexicon	Analysis of deictic, conventional, and representational gestures during mother–infant play session at 18 months Clinical criteria: -MB-CDI-WS long form (American and Italian versions) at 18, 24, 30, 36 months -Mullen Scales of Early Learning (Receptive and/or Expressive Language subscales, American version)
Suttora et al., 2020 [77]	Observational, cross-sectional; clinical setting	N = 61 LT (26 Low-risk preterm; 35 Full Term)	30	Italian	-Expressive lexicon -Expressive grammar	-Parental and child speech collected during a video-recorded 10-min parent–child shared book reading session. Measures: Word types, word tokens, MLU Clinical criteria: -MacArthur–Bates Communicative Development Inventories (MB-CDI) Short Form (Italian version)
Tomas et al., 2015 [92]	Observational, cross-sectional; clinical setting	N = 30 DLD	54–71	Australian English	-Expressive grammar -Non-word repetition	-Question–answer elicitations of the 30 target items, presented along with picture props Clinical criteria: -PLS—Preschool Language Scale -CELF—Clinical Evaluation of Language Function -articulatory screening (non-word repetition)
Vehkavuori and Stolt, 2018 [83]	Observational, cross-sectional; home/clinical setting	N = 78 TLD	24	Finnish	-Communication and symbolic abilities -Receptive and expressive lexicon	-MacArthur Communicative Development Inventories (FinCDI-SF) -Communication and Symbolic Behavior Scales, Developmental Profile, Infant-Toddler Checklist (FinCSBS) Clinical criteria: -Reynell Developmental Language Scales III (Finnish version)

Note. TD—typically developing children; DLD—children with developmental language disorder; LT—late talkers; LD—children with language delay; ASD—Autism Spectrum Disorders.
